# Supplementary material for: Finding Candida auris in public metagenomic repositories
Source: PLoS One. 2024 Jan 19;19(1):e0291406. doi: 10.1371/journal.pone.0291406 (PMC10798454; doi:10.1371/journal.pone.0291406)
Supplement: S1 Table — (DOCX) [file pone.0291406.s001.docx]

S1 Table. **Reference Genomes**. Isolate and clade are only indicated for *C. auris* assemblies.

| GenBank accession | Taxonomic Reference | Isolate | Clade |
| --- | --- | --- | --- |
| GCA_002759435.2 | *Candida auris* | B8441 | 1 |
| GCA_016772235.1 | *Candida auris* | B13916 | 1 |
| GCA_016772135.1 | *Candida auris* | B11205 | 1 |
| GCA_003013715.2 | *Candida auris* | B11220 | 2 |
| GCA_016495645.1 | *Candida auris* | B12043 | 2 |
| GCA_016495685.1 | *Candida auris* | B11809 | 2 |
| GCA_016495665.1 | *Candida auris* | B13463 | 2 |
| GCA_016772175.1 | *Candida auris* | B17721 | 3 |
| GCA_002775015.1 | *Candida auris* | B11221 | 3 |
| GCA_016772215.1 | *Candida auris* | B12037 | 3 |
| GCA_016772195.1 | *Candida auris* | B12631 | 3 |
| GCA_008275145.1 | *Candida auris* | B11245 | 4 |
| GCA_016772155.1 | *Candida auris* | B12342 | 4 |
| GCA_016809505.1 | *Candida auris* | B18474 | 5 |
| GCA_003706955.2 | *Candida blattae* |  |  |
| GCA_002926085.1 | *Candida duobushaemulonis* |  |  |
| GCA_003707735.1 | *Candida freyschussii* |  |  |
| GCA_002926055.1 | *Candida haemuloni* |  |  |
| GCA_003708405.1 | *Candida heveicola* |  |  |
| GCA_003706695.2 | *Candida incommunis* |  |  |
| GCA_001599135.1 | *Candida intermedia* |  |  |
| GCA_900106115.1 | *Candida intermedia* |  |  |
| GCA_900106125.1 | *Candida intermedia* |  |  |
| GCA_003707785.2 | *Candida oregonensis* |  |  |
| GCA_002933435.1 | *Candida pseudohaemulonis* |  |  |
| GCA_003013735.1 | *Candida pseudohaemulonis* |  |  |
| GCA_003243815.1 | *Candida sake* |  |  |
| GCA_000150115.1 | *Ajellomyces capsulatus (Histoplasma capsulatum)* |  |  |
| GCA_000151005.2 | *Ajellomyces capsulatus (Histoplasma capsulatum)* |  |  |
| GCA_000151035.1 | *Ajellomyces capsulatus (Histoplasma capsulatum)* |  |  |
| GCA_000003525.2 | *Blastomyces dermatitidis* |  |  |
| GCA_003206845.1 | *Blastomyces emzantsi* |  |  |
| GCA_000003855.2 | *Blastomyces gilchristii* |  |  |
| GCA_002572885.1 | *Blastomyces parvus* |  |  |
| GCA_001014755.1 | *Blastomyces silverae* |  |  |
| GCA_000149445.2 | *Candida albicans* |  |  |
| GCA_000182965.3 | *Candida albicans* |  |  |
| GCA_000447555.1 | *Candida albicans* |  |  |
| GCA_000447615.1 | *Candida albicans* |  |  |
| GCA_000691765.2 | *Candida albicans* |  |  |
| GCA_000773755.1 | *Candida albicans* |  |  |
| GCA_000774085.1 | *Candida albicans* |  |  |
| GCA_000775465.1 | *Candida albicans* |  |  |
| GCA_000784505.1 | *Candida albicans* |  |  |
| GCA_000784525.1 | *Candida albicans* |  |  |
| GCA_000784575.1 | *Candida albicans* |  |  |
| GCA_003454735.1 | *Candida albicans* |  |  |
| GCA_005890695.1 | *Candida albicans* |  |  |
| GCA_005890745.1 | *Candida albicans* |  |  |
| GCA_005890765.1 | *Candida albicans* |  |  |
| GCA_005890775.1 | *Candida albicans* |  |  |
| GCA_003706475.2 | *Candida corydali* |  |  |
| GCA_000026945.1 | *Candida dubliniensis* |  |  |
| GCA_000002545.2 | *Candida glabrata* |  |  |
| GCA_900535975.1 | *Candida hispaniensis* |  |  |
| GCA_008904905.1 | *Candida metapsilosis* |  |  |
| GCA_000315875.1 | *Candida orthopsilosis* |  |  |
| GCA_000182765.2 | *Candida parapsilosis* |  |  |
| GCA_001442715.1 | *Candida sojae* |  |  |
| GCA_000223465.1 | *Candida tenuis* |  |  |
| GCA_000006335.3 | *Candida tropicalis* |  |  |
| GCA_003707795.1 | *Clavispora fructus* |  |  |
| GCA_000003835.1 | *Clavispora lusitaniae (Candida lusitaniae)* |  |  |
| GCA_001673695.2 | *Clavispora lusitaniae (Candida lusitaniae)* |  |  |
| GCA_003675775.1 | *Clavispora lusitaniae (Candida lusitaniae)* |  |  |
| GCA_009498055.1 | *Clavispora lusitaniae (Candida lusitaniae)* |  |  |
| GCA_000149335.2 | *Coccidioides immitis* |  |  |
| GCA_000149895.1 | *Coccidioides immitis* |  |  |
| GCA_004115165.2 | *Coccidioides immitis* |  |  |
| GCA_000150055.1 | *Coccidioides posadasii* |  |  |
| GCA_000150185.1 | *Coccidioides posadasii* |  |  |
| GCA_000151335.1 | *Coccidioides posadasii* |  |  |
| GCA_000170175.2 | *Coccidioides posadasii* |  |  |
| GCA_001720205.1 | *Cryptococcus amylolentus* |  |  |
| GCA_009650685.1 | *Cryptococcus cf.* |  |  |
| GCA_001720195.1 | *Cryptococcus depauperatus* |  |  |
| GCA_001720245.1 | *Cryptococcus depauperatus* |  |  |
| GCA_006352305.1 | *Cryptococcus floricola* |  |  |
| GCA_000185945.1 | *Cryptococcus gattii* |  |  |
| GCA_000835755.1 | *Cryptococcus gattii* |  |  |
| GCA_000855695.1 | *Cryptococcus gattii* |  |  |
| GCA_002954075.1 | *Cryptococcus gattii* |  |  |
| GCA_000149385.1 | *Cryptococcus neoformans* |  |  |
| GCA_003011985.1 | *Cryptococcus neoformans* |  |  |
| GCA_010065285.1 | *Cryptococcus neoformans* |  |  |
| GCA_010065295.1 | *Cryptococcus neoformans* |  |  |
| GCA_010233705.1 | *Cryptococcus neoformans* |  |  |
| GCA_006149155.1 | *Cryptococcus wingfieldii* |  |  |
| GCA_002110485.1 | *Emergomyces orientalis* |  |  |
| GCA_001883825.1 | *Emergomyces pasteurianus* |  |  |
| GCA_002572855.1 | *Emmonsia crescens* |  |  |
| GCA_001660665.1 | *Emmonsia sp.* |  |  |
| GCA_000150475.2 | *Paracoccidioides brasiliensis* |  |  |
| GCA_000150735.2 | *Paracoccidioides brasiliensis* |  |  |
| GCA_000150705.2 | *Paracoccidioides sp.* |  |  |
| GCA_003054445.1 | *Pichia kudriavzevii (Candida krusei)* |  |  |
| GCA_001477535.1 | *Pneumocystis jirovecii* |  |  |
| GCA_001600855.1 | *Saitozyma sp.* |  |  |
| GCA_002941045.1 | *Sporothrix schenckii* |  |  |
| GCA_009556855.1 | *Talaromyces marneffei* |  |  |
